# Acute Megakaryoblastic Leukemia with Trisomy 21 and Tetrasomy 21 Clones in a Phenotypically Normal Child with Mosaic Trisomy 21

**DOI:** 10.1155/2020/7813048

**Published:** 2020-03-17

**Authors:** Eric Won, Tanja A. Gruber, Suzanne Tucker, Deborah E. Schiff

**Affiliations:** ^1^Department of Pediatrics, UCSD School of Medicine, La Jolla, CA, USA; ^2^Division of Hematology-Oncology, Rady Children's Hospital, San Diego, CA, USA; ^3^Department of Oncology, St. Jude Children's Research Hospital, Memphis, TN, USA; ^4^Department of Pathology, UCSD School of Medicine, La Jolla, CA, USA; ^5^Division of Pathology, Rady Children's Hospital, San Diego, CA, USA

## Abstract

Pediatric acute megakaryoblastic leukemia (AMKL) is a rare subtype of acute myeloid leukemia (AML) that may be divided into two subgroups: (1) Down syndrome- (DS-) related AMKL which generally has a favorable prognosis and (2) non-DS-related AMKL which generally has a poorer outcome. We report a phenotypically normal child with AMKL with trisomy 21 (T21) and tetrasomy 21 clones. Subsequently, she was diagnosed with mosaic T21. She underwent reduced-intensity therapy with good outcome. We review the literature regarding AMKL-associated cytogenetic abnormalities and AMKL in association with DS. We suggest evaluation for mosaic T21 in phenotypically normal pediatric patients with T21-positive AML.

## 1. Introduction

AMKL is a rare subtype of AML that occurs in approximately 4–15% of newly diagnosed pediatric AML [1, 2]. However, in children with Down syndrome (DS), AMKL is the most frequent type of AML; DS patients have a 500-fold higher incidence of AMKL than the general population [2, 3]. Historically, non-DS-AMKL has been associated with poorer outcomes and requires higher-intensity chemotherapy, while DS-AMKL is associated with a more favorable prognosis despite reduced-intensity regimens [1, 2, 4, 5]. Prior studies have also suggested that patients with mosaic trisomy 21 (T21) and associated AMKL can also receive reduced-intensity regimens with good outcomes [6, 7]. Therefore, it is important to correctly identify patients who have DS-AMKL versus non-DS-AMKL because the optimal therapy intensity for each differs greatly (Table 1). Here, we report a case of AMKL with T21 and tetrasomy 21 clones in a phenotypically normal child who was subsequently diagnosed with mosaic T21. We review the literature regarding AMKL-associated cytogenetic abnormalities and AMKL in association with DS.

## 2. Case Report

A 2-year-old girl presented with a 1-month history of increased fatigue and irritability. She had no significant past medical history. Her developmental history was normal, except for mild speech delay. Her exam revealed no short stature, upslanting eyes, flat nasal bridge, short fingers, low tone, or other phenotypic evidence of DS. Her labs showed pancytopenia (WBC 7.8 TH/*μ*L, Hgb 5.8 g/dL, platelets 129 TH/*μ*L, and ANC 608/*μ*L) with normal uric acid, lactate dehydrogenase, and iron studies, and no peripheral blasts. Chest X-ray was normal.

**Figure 1:**
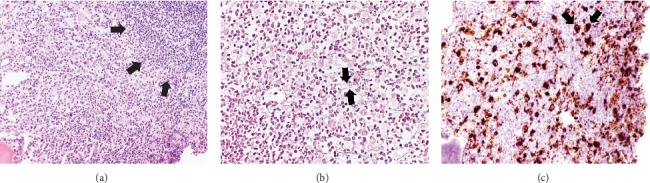
(a) Hypercellular bone marrow core biopsy with lymphocytic infiltrate (arrows) and a few dysplastic cells (hematoxylin-eosin). (b) Reticulin stain demonstrates increased reticulin fibers (arrows). (c) Immunohistochemical staining for CD61 reveals numerous CD61-positive immature cells with megakaryocytic differentiation (arrows).

Bone marrow aspiration and biopsy and lumbar puncture were performed. CSF cytology was negative for malignant cells. Flow cytometry of her bone marrow aspirate (BMA) showed no immunophenotypic abnormalities and consisted primarily of maturing hematopoietic cells with lymphocytes (T-cells and polyclonal B-cells) and a small population of B-cells coexpressing CD19/CD10 (8%), most consistent with hematogones. BMA differential showed 3% blasts. The cellularity of the BMA smears was low. No distinct marrow particles were seen. The hematopoietic cells were predominately composed of lymphoid precursors admixed with scattered erythroid and myeloid derivatives. No dysplasia was identified. Occasional hematogones were present. Clusters of tumor cells were not noted. However, her bone marrow core biopsy was hypercellular (>95% cellularity) with a few dysplastic cells (Figure 1(a)). Reticulin stain revealed increased reticulin fibers (Figure 1(b)). Because the BMA and biopsy were nondiagnostic, immunostaining for megakaryocytic marker CD61 was performed on the bone marrow core and revealed numerous (>20%) CD61-positive immature cells that demonstrated histologic megakaryocytic differentiation and appeared in sheets (Figure 1(c)). The morphological features of the core biopsy combined with the results from CD61 immunostaining yielded the diagnosis of AMKL.

FISH performed on BMA showed two populations of cells—T21 (13%) and tetrasomy 21 (9%). FISH was negative for the following: deletion 9p21; t (9; 22); rearrangements of MYC, MLL, IGH, E2A, and GLIS2 loci and cytogenetic abnormalities commonly observed in myeloid neoplasms. RNA sequencing to identify AML-associated fusion genes was negative for the following: DEK-NUP214 [t(6;9)], KAT6A-CREBBP [t(8;16)], −7, −5, 5q, KMT2A-MLLT10 [t(6;11)], KMT2A-MLLT4 [t(10;11)], inv(3)(q21q26.2), CBFA2T3-GLIS2 [inv(16)(p13.3q24.3)], NUP98-KDM5A [t(11;12)(p15;p13)], ETV6-HLXB [t(7;12)(q36;p13)], NUP98-HOXA9 [t(7;11)(p15.4;p15)], NUP98-NSD1. FLT3-ITD was not detected by PCR. GATA1 mutations were not detected by targeted Sanger sequencing; WT1 mutations were not detected by targeted NGS.

Once diagnosed with AMKL, IRB consent was obtained, and she was treated on SJCRH protocol AML16 (NCT03164057). She received epigenetic priming with azacitidine (75 mg/m^2^/day IV on days −4 to 0), followed by induction therapy with cytarabine (100 mg/m^2^/q12 h IV on days 1–10), daunorubicin (50 mg/m^2^/day IV on days 1, 3, and 5) with dexrazoxane as a cardioprotectant, and etoposide (100 mg/m^2^/day IV on days 1–5). By day 22 of induction, her BMA and biopsy were negative for malignant cells by morphology, and the tetrasomy 21 clone was undetectable. However, T21 was still present in 11% of cells. Due to concern that the eradicated tetrasomy 21 clone represented treated AMKL blasts and that she could have mosaic T21 despite the lack of phenotypic evidence of DS, she underwent a skin biopsy/fibroblast culture for FISH screening which showed T21 in 86% of fibroblasts, thus confirming the diagnosis of mosaic T21.

Her therapy was changed from intensive non-DS-AML therapy to reduced-intensity chemotherapy for DS-AML according to COG protocol AAML1531 (NCT02521493) Standard Risk arm with one additional cycle of cytarabine and asparaginase as recommended in COG AAML1531 memo dated 10/18/2018 [8]. Her total dose of cytarabine, daunorubicin equivalent, etoposide, and the number of chemotherapy cycles are reported in Table 1 and compared with non-DS-AMKL and DS-AMKL therapy.

She tolerated therapy well, with two episodes of grade 3 bacteremia and without grade 4 nonhematologic complications. At the last medical follow-up, she was off therapy for 11 months and doing well without evidence of treatment-related toxicity or relapse.

## 3. Discussion

In this report, we present a case of AMKL in a patient with mosaic T21. Approximately 1.3–5% of people with Down syndrome have mosaic T21 [9]. Based on population studies, approximately 37.5% of individuals with T21 mosaicism were detected by physical examination alone compared to 100% of individuals with nonmosaic T21 [9]. Cases have been reported of pediatric AMKL with GATA1 mutations which led to the diagnosis of previously unrecognized mosaic T21; a subset of these patients had normal phenotypes [9]. Due to the inability to exclude mosaic T21 by physical examination alone and the low frequency of somatic T21 in pediatric non-DS-AML [10], we recommend that patients without known DS but with T21-positive AML should undergo an evaluation for germline T21 via skin biopsy, even in the absence of phenotypic features of DS. It is important to distinguish DS-AML from non-DS-AML because outcomes for DS-AML are better, in general, than for non-DS-AML, and patients with DS-AML may receive less intensive therapy with excellent outcomes [5–7]. Furthermore, patients with DS have increased risk for treatment-related toxicity; high-intensity therapy unnecessarily increases treatment-related toxicity and mortality. As shown in Table 1, this child with AMKL and mosaic T21 was able to receive lower cumulative doses of anthracycline and cytarabine without compromising treatment outcome.

AMKL is caused by a heterogeneous group of mutations [2, 4, 11]. Based on these mutations, pediatric AMKL may be stratified into high-risk and standard-risk groups. NUP98/KDM5A, CBFA2T3/GLIS2, KMT2A-rearranged lesions, and monosomy 7 (NCK-7) independently predict a poor outcome, and AMKL patients with these genetic alterations should receive intensified therapy [4]. Non-DS-AMKL patients with RBM15/MKL1 fusion have a good prognosis and may receive standard-intensity therapy if their AMKL displays a good response to induction therapy. DS-AMKL, characterized by GATA1 mutations, has a good prognosis when treated with reduced-intensity chemotherapy. The rearrangements commonly seen in non-DS-AMKL (RBM15/MKL1, CBFA2T3/GLIS2, NUP98/KDM5A, and KMT2A rearrangements) do not occur with DS-AMKL [2, 4, 5, 12, 13]. Although GATA1 mutations are usually associated with DS-AMKL, there have been rare cases of non-DS-AMKL with GATA1 mutations, usually in association with acquired T21. These patients also appear to have a favorable prognosis [2, 4].

AMKL is often associated with myelofibrosis, which may delay diagnosis due to difficulty in obtaining sufficient leukemia cells by bone marrow aspiration. In the setting of myelofibrosis, an assessment for immunophenotypic, cytogenetic, and genetic abnormalities by bone marrow aspiration may yield false-negative results. In this case report of a child with mosaic T21 and DS-AMKL, flow cytometry performed on BMA showed no immunophenotypic abnormalities, morphological review of BMA revealed no clusters of tumor cells, and BMA failed to show the expected GATA1 mutation. The above negative findings can be attributed to low leukemia burden in the BMA. Of note, GATA1 sequence variants present in less than 50% of a patient's nucleated cells may not be detected by the targeted Sanger sequencing method used to evaluate this child's BMA for a GATA1 mutation. To facilitate the diagnosis of suspected AMKL, we recommend immunostaining the bone marrow core biopsy for megakaryocytic markers CD42b and/or CD61 [14]. In this case, CD61 immunostaining confirmed AMKL.

In summary, this case highlights the importance of testing for mosaic T21 in children with AML and T21-positive clones. The absence of classic phenotypic DS features alone is not sufficient to exclude mosaic T21. Pediatric patients with mosaic T21 and AML may receive a reduced-intensity regimen with good outcomes and decreased treatment-related morbidity and mortality.

## Figures and Tables

**Table 1:** Comparison of modified therapy for child with mosaic T21 with AML16 (NCT03164057) for non-DS-AML and AAML0431 (NCT00369317) for DS-AML.

	AML16 for non-DS-AML	Modified AMKL therapy for child with mosaic T21	AAML0431 for DS-AML
Cytarabine gm/m^2^	54	28.6	27.8
Daunorubicin equivalent mg/m^2^	414	310	240
Etoposide mg/m^2^	1250	1250	750
Total number of cycles	5	6	6

## Abbreviations

AMKL: Acute megakaryoblastic leukemia
AML: Acute myeloid leukemia
DS: Down syndrome
T21: Trisomy 21
Non-DS-AMKL: AMKL in patients without Down syndrome
DS-AMKL: AMKL in patients with Down syndrome
Non-DS-AML: AML in patients without Down syndrome
DS-AML: AML in patients with Down syndrome
WBC: White blood cell count
Hgb: Hemoglobin
ANC: Absolute neutrophil count
CSF: Cerebrospinal fluid
FISH: Fluorescent in situ hybridization.
